# Retraction: To Study the Effect of Anterior Palatal Surface Modifications of Complete Denture on Speech Intelligibility Oral Perception and Cortical Brain Function Activity: An In Vivo Study

**DOI:** 10.7759/cureus.r71

**Published:** 2023-05-18

**Authors:** Prince Kumar, Challagondla Bhargavi, Shrinivas Dharaskar, Nikhat Fatima, Manjiri Salkar, Ashwini Dhopte

**Affiliations:** 1 Prosthodontics, Rama Dental College, Hospital and Research Centre, Kanpur, IND; 2 Prosthodontics, Mamata Dental College and Hospital, Khammam, IND; 3 Prosthodontics, ACPM Dental College, Dhule, IND; 4 Periodontology, Rama Dental College, Hospital and Research Centre, Kanpur, IND; 5 Prosthodontics, Mahatma Gandhi Vidyamandir’s KBH Dental College and Hospital, Nashik, IND; 6 Oral Medicine and Radiology, Rama Dental College and Research Centre, Kanpur, IND

This article has been retracted due to plagiarism. The journal was contacted by the author of the following PHD thesis (published in 2021) and corresponding article (published in 2018) with allegations that the article published in Cureus has been plagiarized from these sources:

- https://shodhganga.inflibnet.ac.in/handle/10603/247044 To study the effect of anterior palatal surface modifications of complete denture on speech intelligibility oral perception and cortical brain function activity an in vivo study
- https://www.jcdr.net/ReadXMLFile.aspx?id=11556 The Effect of Anterior Palatal Surface Modifications of Complete Denture on Speech Intelligibility

The submitting author has admitted to this plagiarism and also requested retraction.

